# The Role of Biological Effective Dose in Gamma Knife Radiosurgery: A Systematic Review Across Multiple Indications

**DOI:** 10.3390/jcm15010381

**Published:** 2026-01-05

**Authors:** Hao Deng, Xinyuejia Huang, Qian Wang, Yuan Gao, Mengqi Wang, Yang Wu, Xiaoman Shi, Maoyu Wang, Wei Pan, Senlin Yin, Wei Wang

**Affiliations:** 1 Department of Neurosurgery, West China Hospital of Sichuan University, 37 Guoxue Alley, Chengdu 610041, China; 2023324025260@stu.scu.edu.cn (H.D.); hxyj2023@stu.scu.edu.cn (X.H.); gaoyuan_scu@wchscu.cn (Y.G.); wmq_1101@scu.edu.cn (M.W.); 2023224025415@stu.scu.edu.cn (X.S.); 18011376726@163.com (W.P.); 2 Department of Respiratory and Critical Care Medicine, West China Hospital, Chengdu 610041, China; wq1996wcrm@foxmail.com

**Keywords:** Gamma knife radiosurgery, biological effective dose, intracranial tumor, arteriovenous malformation, trigeminal neuralgia, essential tremor

## Abstract

**Background:** Gamma Knife radiosurgery (GKS) is widely used for the management of intracranial disorders. Emerging evidence suggests that incorporating the biological effective dose (BED) into GKS planning may improve the prediction of treatment efficacy and toxicity. This review aims to evaluate the role of BED in GKS across multiple intracranial indications. **Methods:** A qualitative review of published clinical studies was performed to assess the application of BED models in GKS for pituitary adenomas, vestibular schwannomas, meningiomas, arteriovenous malformations (AVMs), trigeminal neuralgia, and other disorders. The relationships between BED, treatment outcomes, and adverse effects were compared across indications. **Results:** The association between BED and clinical outcomes was most consistent in AVMs, where higher BED correlated closely with obliteration rates. In other diseases, BED-based analyses showed promising but variable predictive value. Notably, BED-derived parameters demonstrated improved prediction of post-GKS hypopituitarism in pituitary adenomas and AVM obliteration compared with physical dose alone. However, most available evidence was derived from retrospective studies. **Conclusions:** BED may serve as a valuable complement to conventional physical dose metrics in GKS planning, but its ability to replace physical dose remains uncertain. Prospective studies and histology-specific radiobiological parameter validation are required to establish the routine clinical utility of BED.

## 1. Introduction

Gamma knife radiosurgery (GKS) is a widely used radiotherapy technique for treating intracranial disorders. It is now indicated for a large variety of diseases, including pituitary tumors, vestibular schwannomas, arteriovenous malformations (AVMs), meningiomas, metastases, and functional disorders [1,2,3,4,5]. GKS delivers a single, high-dose radiation treatment with submillimeter precision, which minimizes damage to adjacent healthy tissue [1]. Although previous studies have identified the recommended range of physical doses for various indications, determining the optimal radiation dose to maximize treatment efficacy while minimizing adverse effects remains a significant challenge.

Increasing evidence suggests that incorporating the biological effective dose (BED) into GKS treatment plans may enhance outcomes [6,7]. The concept of BED, introduced in 1989, quantifies the biological effectiveness of radiotherapy treatments [8], accounting for cellular DNA repair during radiation exposure [9]. While BED has been widely used in conventional fractionated radiotherapy, a model suitable for BED calculations in GKS was not proposed until recent years [10]. Moreover, rising interest in dose-rate modeling, the early integration of radiobiological parameters into contemporary treatment-planning systems, and the increasing availability of longitudinal outcome datasets have collectively renewed attention to BED-based approaches in GKS. These developments provide an opportunity to reassess how biological dose modeling may enhance treatment. Interest in integrating BED into radiosurgery planning has grown, with several studies examining its effects on GKS across various diseases [11,12,13]. However, no prior work has systematically summarized the evidence on BED across multiple GKS indications using a consistent radiobiological framework. To address this gap, we conducted a comprehensive literature review to evaluate the relationship between BED and treatment outcomes across diverse GKS applications, aiming to clarify the current evidence base and identify future directions for integrating BED into radiosurgery practice.

## 2. Methods

The PubMed-Medline, Ovid-Embase, and Cochrane Library databases were searched on 23 September 2025. The search strategy used the following terms: [“Gamma Knife” OR “GKS” OR “radiosurgery”] AND [“biological effective dose” OR “BED” OR “biologically effective dose”]. Only literature published in English was included.

The inclusion criteria were as follows: (a) GKS was performed for neurosurgical or neurofunctional diseases, (b) the effects of BED on the efficacy and/or safety outcomes of GKS were reported, and (c) BED calculation was based on the biexponential DNA repair model. While the traditional linear-quadratic (LQ) model is commonly used to estimate BED, it assumes a single exponential repair process and neglects sublethal damage repair during irradiation [14]. This simplification may not be suitable for GKS, where high-dose radiation is delivered over extended time periods [15]. In contrast, the biexponential DNA repair model accounts for both fast and slow repair kinetics, offering a more accurate representation of biological response by considering ongoing DNA repair during treatment [16]. Therefore, only studies that employed the biexponential model for BED estimation were included in this review. The BED formulation was presented in Supplementary Materials.

The exclusion criteria were: (a) full text was unavailable, (b) reviews, meta-analyses, conference abstracts, or commentary letters were excluded, and (c) studies that did not describe the BED model were excluded.

**Figure 1:**
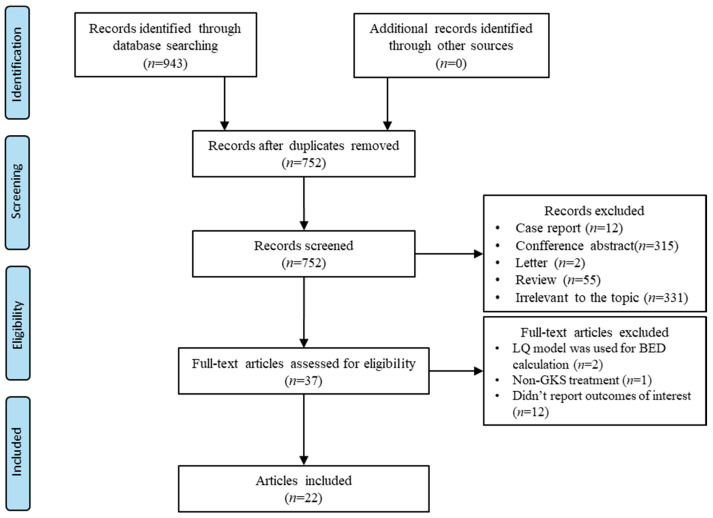
Flow diagram summarizing the process of literature selection.

Literature selection and data extraction were performed independently by two reviewers (H.D., Q.W.). The reviewers screened the initial literature by reading the titles and abstracts. Eligible studies were then further evaluated through full-text reading by the same two reviewers. Discrepancies were first addressed through structured discussion to reach consensus; if disagreements persisted, a senior author (W.W. or S.Y.) served as an adjudicator to provide the final decision. The senior authors (W.W., S.Y.) were consulted when any inconsistencies and conflicts emerging. For each included study, the following data were extracted: the first author’s name, publication year, study type, baseline patient characteristics, disease type, GKS techniques, physical dose, BED, and other relevant data. Based on the GKS indications covered in the included studies, we collected all research findings related to the efficacy and safety of GKS, including but not limited to biochemical remission of pituitary tumors, changes in tumor volume, AVM obliteration, relief of trigeminal neuralgia, new-onset hypopituitarism, hearing loss, radiation-induced brain edema, and facial sensory loss. To assess the methodological quality and risk of bias of the included observational studies, we applied an adapted version of the Newcastle–Ottawa Scale (NOS), evaluating study selection, comparability, and outcome ascertainment. The results of this assessment are summarized in Supplementary Table S1. A total of 22 studies were included in this review. The selection process is depicted in the flow chart in Figure 1. The diseases covered in these studies included pituitary adenoma (*n* = 6) [12,17,18,19,20,21], meningioma (*n* = 3) [22,23,24], vestibular schwannomas (*n* = 4) [13,25,26,27], arteriovenous malformations (AVM) (*n* = 3) [7,11,28], melanoma brain metastases (*n* = 1) [29], trigeminal neuralgia (*n* = 4) [6,30,31,32], and essential tremor (*n* = 1) [33]. The characteristics of included studies are listed in Table 1. The Effects of BED and physical dose on treatment outcomes are presented in Table 2. This review was not registered in PROSPERO or other systematic review registries, which should be considered a methodological limitation. Table S3: PRISMA 2020 Checklist see the Supplementary Materials.

## 3. Results

### 3.1. Pituitary Adenoma

Among the studies that included pituitary adenoma, three focused on acromegaly, two on Cushing’s disease, and one on multiple pituitary adenomas. For acromegaly, the results from different centers were generally consistent. Two studies identified BED as a significant predictor of biochemical remission after GKS. Dumot et al. reported that the probability of biochemical remission in patients receiving a BED > 170 Gy_2.47_ was twice that of those receiving a lower BED (HR: 2.02, 95% CI: 1.06–3.86, *p* = 0.03) [21]. In a study by Graffeo et al., BED was significant for biochemical remission both as a continuous variable (HR: 1.01, 95% CI: 1.00–1.02, *p* = 0.02) and as a binary variable (BED > 200 Gy_2.47_, HR: 2.72, 95% CI: 1.36–5.13, *p* < 0.01) [17]. Another study showed that the higher BED group tended to have better endocrine remission at 9 years (70.2% vs. 48.2%, *p* > 0.05) [18]. Although the difference did not reach statistical significance, this qualitative trend aligned with the findings of the other two studies. Neither BED nor physical dose was significantly associated with post-GKS hypopituitarism.

For Cushing’s disease, both studies had rather limited sample sizes. Our center reported a significantly higher endocrine remission rate in the higher BED group (>205 Gy_2.47_) at 2 years after GKS (72.7% vs. 35.1%, *p* = 0.04) [12]. Balossier et al. presented a similar trend, though the difference was not statistically significant [19]. Graffeo et al. included a cohort with both secreting and non-secreting tumors. Their results indicated that mean gland BED, rather than BED alone, was significantly associated with post-GKS hypopituitarism (HR: 1.03, 95% CI: 1.02–1.05, *p* < 0.001) [20]. However, neither physical dose nor BED nor mean gland BED were associated with treatment success. Additionally, although mean gland dose was a predictor of post-GKS hypopituitarism (HR: 1.31, 95% CI: 1.16–1.47, *p* < 0.001), the BED-based model demonstrated better predictive efficacy compared to physical dose-based models.

### 3.2. Meningioma

Huo et al. reported the largest meningioma cohort (n = 336) among the included studies [23]. The effects of BED on local tumor control varied across different WHO grades. In a cohort of 354 grade I lesions, a BED > 50 Gy_2.47_ was significantly associated with a lower incidence of local failure (*p* < 0.01). Similarly, another study involving 91 cases of parasellar meningioma (WHO grade I or presumed grade I) also found that BED was a significant predictor of local control (HR: 0.96, 95% CI: 0.92–1.00, *p* = 0.03) [24]. However, Dedeciusova et al. reported mixed results, finding no significant association between BED and clinical improvement or tumor control [22]. Their study, which included only 46 cases, may have lacked sufficient statistical power to detect a significant effect. Notably, none of the included studies addressed the impact of BED on the safety of GKS.

### 3.3. Vestibular Schwannomas

The studies on vestibular schwannomas showed heterogeneous results. The largest cohort study failed to find a significant association between BED and either tumor control or symptomatic edema [25]. However, Tuleasca et al. reported a significantly positive association between BED and tumor volume changes (β = −0.17, *p* < 0.01) [13]. Additionally, Tuleasca et al. explored the relationship between BED at specific sites and hearing decline. Both tumor BED and mean cochlea BED were significantly related to hearing decline at various time points [26,27]. Interestingly, mean cochlea dose, rather than tumor dose, was significantly associated with hearing decline at 24 months.

### 3.4. Arteriovenous Malformation

All three included studies indicated that BED was a significant predictor of AVM obliteration after GKS. Nesvick et al. reported that the probability of obliteration in patients with a BED > 133 Gy_2.47_ increased by 52% compared to those receiving a lower BED (HR: 1.52, 95% CI: 1.19–1.95, *p* < 0.01) [11]. In another study, patients with a BED > 180 Gy_2.47_ had more than twice the probability of obliteration (HR: 2.11, 95% CI: 1.30–3.40, *p* < 0.01) [28]. Tuleasca et al. developed a predictive model for AVM obliteration after GKS, which showed that BED was the strongest predictor of obliteration in unruptured AVMs (HR: 1.015, 95% CI: 1.001–1.029, *p* = 0.03) [7]. Additionally, they found that BED had greater predictive power than physical dose. Although Nesvick et al. reported on the largest cohort, their study did not address the effects of BED on the safety of GKS. The other two studies showed that neither BED nor physical dose were significantly associated with post-GKS complications.

### 3.5. Trigeminal Neuralgia and Other Indications

Tuleasca et al. reported the earliest results of BED on TN. They observed a trend of increasing hypoesthesia with higher BED, but did not find a definitive relationship between BED and pain relief [30]. The following studies showed that effects of BED on TN may vary for different trigeminal nerve target. A multicenter cohort including 871 cases of type 1 TN showed that a BED ≥ 2100 Gy_2.47_ was significantly associated with initial pain relief for the distal target (HR: 1.46, 95% CI: 1.05–2.03, *p* = 0.03) [6]. For the proximal target, physical dose rather than BED was a significant predictor of initial pain relief (HR: 1.79, 95% CI: 1.05–3.05, *p* = 0.03). However, neither BED nor maximal dose was associated with post-GKS sensory dysfunction for either proximal or distal target. Similarly, results of our center also showed target heterogeneity of BED [32]. For patients with distal targets, BED was a significant predictor of treatment failure (OR: 0.996, 95% CI: 0.992–0.999, *p* = 0.02) and post-GKS complications (OR: 1.002, 95% CI: 1.000–1.004, *p* = 0.01). BED did not significantly influence outcomes in the proximal target subgroup, either for treatment failure or complications. Tang et al. combined BED with target volumes to explore the association between BED coverage and treatment outcomes [31]. The results indicated that V%_CN V-BED1000_, rather than BED alone, was a significant predictor of pain relief (OR: 1.05, 95% CI: 1.04–1.07, *p* < 0.01), quality of life (OR: 1.05, 95% CI: 1.04–1.07, *p* < 0.01), and medication withdrawal (OR: 1.05, 95% CI: 1.04–1.05, *p* < 0.01). Additionally, the maximal BED received by the brainstem was a significant predictor of post-GKS complications (OR: 1.06, 95% CI: 1.05–1.06, *p* < 0.01).

Only one study reported results on essential tremor (ET) [33]. Increasing BED was associated with improvement in the ET rating scale (β = −0.029, *p* = 0.04). Adverse radiation events tended to occur in cases receiving high BED (>4500 Gy_2.47_). Zubatkina et al. used multiple α/β ratios (3, 5, 10, 15) to calculate BED in melanoma brain metastases [29]. BED, based on various α/β ratios, was significantly associated with local control after GKS. Furthermore, BED calculated with an α/β ratio of 15 demonstrated better predictive efficacy for local control compared to the margin dose (AUC: 0.85 vs. 0.79). Neither BED nor margin dose was significantly associated with radiation necrosis. The interpretive strength of findings for essential tremor and melanoma brain metastases is limited, as each indication was represented by only a single eligible study. Consequently, conclusions for these categories should be regarded as preliminary, and additional standardized, high-quality investigations are required to validate these observations.

## 4. Discussion

Our review indicates that BED may play a meaningful role in influencing clinical outcomes across a variety of GKS indications. The most consistent findings were observed for AVMs, where all studies identified BED as a significant predictor of obliteration. Results for pituitary adenomas also showed a relatively coherent pattern, with multiple studies demonstrating associations or trends between BED and endocrine remission, while mean gland BED emerged as a predictor of post-GKS hypopituitarism. In contrast, for indications such as TN, meningioma, and VS, study findings were more variable, likely reflecting differences in target selection, coverage metrics, and study methodology. The limited evidence available for essential tremor and melanoma brain metastases should be interpreted cautiously, as each was represented by a single study. We have summarized the indication-specific BED thresholds in the Supplementary Table S2.

Early radiobiological work quickly recognized that treatment outcomes depend not only on the physical dose but also on its biological effectiveness [14]. Currently, BED is widely applied in conventional fractionated radiotherapy, where numerous clinical series have linked higher BED values to improved tumor control and survival across various diseases [34,35,36]. However, unlike conventional radiotherapy, GKS delivers a very high dose through single or multiple isocenters over a long period of time. This generates steep intracranial dose gradients, allowing DNA repair to begin during—rather than after—irradiation. A variety of studies suggest that the administration of a single, high dose of radiation in vivo has a much greater effect than that which would be predicted from the LQ model using the coefficients calculated from conventional in vitro dose/fractions [15]. Modeling studies have shown that the LQ-based BED can misestimate biological effects in GKS [37,38]. To address this, Jones et al. introduced a biexponential, time-corrected BED framework for GKS, which explicitly accounts for both fast and slow DNA repair kinetics during protracted single-session treatments [10]. All studies included adopted this BED model, which helped reduce bias and heterogeneity.

The physical dose used in GKS varied substantially across indications, and dose selection is still largely empirical. Due to the varying dose rate of Co^60^, the delivery time for a given physical dose can differ considerably. Sublethal DNA damage begins to repair while irradiation is still ongoing [10]. Therefore, the same prescribed dose in GKS may produce different biological effects, especially when the dose rate varies widely. Some studies have explored the impact of dose rate. Lee et al. and Barzaghi et al. reported that a higher dose rate was associated with a lower likelihood of recurrence for TN [39,40]. Huo et al. found that meningioma patients treated with lower dose rates had a higher incidence of local failure compared to those treated with higher dose rates. However, Tuleasca et al. found no significant association between dose rate and AVM obliteration or post-SRS complications [7]. The introduction of BED in GKS allows for dose–time compensation that physical dosimetry fails to account for. Notably, three studies directly compared BED with physical dose and showed that BED provided superior predictive performance for hypopituitarism, AVM obliteration, and local control in melanoma brain metastases [7,20,29]. This potential advantage suggests that clinicians may benefit from consciously incorporating BED into GKS planning. Some low-level evidence even suggested a specific BED range for certain indications, though these BED ranges require further validation [21,33]. Additionally, BED may offer personalized dose constraints for organs at risk. For example, incorporating BED into planning has been shown to refine cochlear and brainstem limits and correlate more closely with hearing preservation outcomes after vestibular schwannoma GKS than physical dose alone [27].

Several studies highlighted that BED-derived parameters such as volume-based BED coverage and mean gland BED, may better characterize the biological distribution of dose than gross BED [20,31]. For instance, the use of mean gland BED rather than gross lesion BED underscores the differing radiosensitivities of surrounding anatomical structures and reinforces the importance of sparing critical tissues during GKS planning. Likewise, volume-based BED coverage reflects not only the existence of an effective BED threshold but also the necessity of achieving adequate biological dose coverage across the target, combining both dose adequacy and spatial distribution into a single metric. Together, these multidimensional BED-derived parameters may offer a potentially more predictive framework than gross BED alone, although further validation is still required. Current commercial GKS planning systems do not natively compute BED, but BED-based corrections can be implemented via exported dose-rate data, custom calculation modules, or research-grade plugins. Incorporating BED requires familiarity with radiobiological modeling, which introduces a learning curve for clinicians. However, the increasing availability of automated BED calculators and scripting interfaces makes that practical implementation is feasible.

One of the limitations is that the same radiobiological parameters were used for BED estimation across different histological types. For instance, the α/β ratio for normal brain tissue was assumed to be 2.47 Gy [41], and all studies, except for that on melanoma brain metastases, used 2.47 Gy as the α/β ratio. While this approach serves as a compromise due to the current lack of histology-specific parameters, it introduces potential systematic errors when applying BED in GKS. According to the BED formulation, Underspecifying α/β leads to an overestimation of BED, whereas overspecifying it causes underestimation. Using a uniform α/β ratio of 2.47 Gy across diverse histologies may distort the magnitude of BED, alter BED–outcome correlations, and destabilize disease-specific BED thresholds, underscoring the importance of deriving histology-specific radiobiological parameters. Zubatkina et al. explored various α/β ratios in BED estimation and found that α/β ratios of 10 Gy and 15 Gy might be more appropriate for melanoma brain metastases based on clinical outcomes [29]. Reverse outcome-based modeling may therefore offer a feasible and informative strategy for establishing histology-specific radiobiological parameters. However, such analyses require large, well-characterized cohorts with standardized definitions of endpoints and harmonized dose-time reporting, necessitating multi-center collaboration. Importantly, clinically derived α/β ratios should ideally be cross-validated with parameters obtained from biological experiments, which may help refine BED estimation and improve the robustness of radiobiological modeling in GKS. Finally, substantial heterogeneity existed across studies. Variability in study design (single-center vs. multicenter cohorts), inconsistency in outcome definitions and the use of different GKS platforms across institutions may all introduce methodological inconsistency. Moreover, marked variation in follow-up duration and baseline characteristics, including age distribution and sex ratio, further complicates direct comparison. These differences may partially explain the inconsistency of findings for certain indications.

Overall, BED appears to be a significant factor across a wide range of GKS indications. This observation suggests that BED-driven optimization could serve as a unifying framework for GKS planning. While integrating BED into treatment planning offers valuable biological insights and may complement traditional physical dose parameters, whether BED can ultimately replace physical dose as the primary planning metric remains uncertain. All of the supporting evidence is retrospective and rarely incorporates prospectively collected data on toxicity or treatment efficacy endpoints. Prospective studies are essential to determine whether BED-optimized plans improve outcomes compared to standard physical dose prescriptions.

## 5. Conclusions

Our review suggests that BED may play a meaningful role in influencing clinical outcomes across a range of GKS indications. Among these, the association between BED and treatment efficacy is most consistent in AVMs, where BED aligns closely with obliteration rates. For other indications, including vestibular schwannoma and meningioma, these results remain more variable, though several studies support the potential utility of BED. While integrating BED into treatment planning offers valuable biological insights and may complement traditional physical dose parameters, whether BED can ultimately replace physical dose as the primary planning metric remains uncertain. Further research is needed to determine histology-specific radiobiological parameters for more precise BED estimation and to validate the routine clinical utility of BED through prospective studies especially those incorporating standardized toxicity endpoints, functional outcome measures, and harmonized radiobiological reporting.

## Figures and Tables

**Table 1:** Characteristics of included studies.

Studies	Disease	Patients	Male (%)	Age (Median, Year)	FU (Median, Month)	Margin Dose (Median, Gy)	BED (Median, Gy_2.47_)
Graffeo et al., 2020 [17]	Acromegaly	102	54.9	49, IQR 37–59	63, IQR 29–100	25, IQR 21–25	169.5, IQR 125–196
Balossier et al., 2021 [18]	Acromegaly	42	52.4	48, range 23–72	60.5, range 9–127	28, range 20–35	199.7, range 64.1–237.1
Dumot et al., 2023 [21]	Acromegaly	67	53.7	46.8, IQR 21.2	66, IQR 80.4	25, IQR 5	171.9, IQR 66.0
Balossier et al., 2021 [19]	Cushing disease	26	76.9	39, range 15.4–69.5	80, range 19–141	27.5, range 24–35	228.1, range 160–248
Gao et al., 2023 [12]	Cushing disease	31	22.6	36, range 20–71	22, IQR 14–34	28, IQR 26–31	221.5, IQR 192.2–259.6
Graffeo et al., 2021 [20]	Non-secreting/secreting adenoma	97	45	50, IQR 38–57	48, IQR 34–68	30/50, IQR 28–32/45–50	NA
Dedeciusova et al., 2022 [22]	Meningioma	46	19.6	59.7, range 31–82	43.8, range 6–108	12.4, range 12–14	60.1, range 49.1–88.3
Huo et al., 2022 [23]	Meningioma	336	26.8	57, range 23–92	41.3	12, range 10–18	60.3, range 30.4–118
Shaaban et al., 2024 [24]	Meningioma	91	22.0	55, range 47.5–65.5	46	14, range 13–15	71.6, range 62–77
Tuleasca et al., 2021 [13]	VS	159	45.3	58.5, range 21.1–83.6	61.2, range 36–110	12 Gy for all	66.3, range 54.1–73.9
Villafuerte et al., 2021 [25]	VS	607	46	59, range 17–90	60	NA	56.9, range 40.1–64.0
Tuleasca et al., 2023, 2024 [26,27]	VS	213	48.4	54, range 21.7–86.1	36, range 6–84	12 Gy in 210 cases 11 Gy in 5 cases	Mean 57.1, range 42.7–66.3
Nesvick et al., 2021 [11]	AVM	352	43.9	39, IQR 27–50	70.8	18.8, IQR 18–20	129.9, IQR 109.15–157.06
Tuleasca et al., 2021 [7]	AVM	149	45	40, range 18–68	48, range 12–154	24 range 18–25	229.9, range 106.7–246.8
Grogan et al., 2024 [28]	AVM	197	54.3	13.1, IQR 5.2	34.2, IQR 2.26	22, IQR 4	183.2, IQR 70.5
Zubatkina et al., 2023 [29]	MBM	73	NA	NA	6.1, range 0.8–49.4	22, range 15–25	103.9 (Gy_5_), 62.7 (Gy_10_), 50.3 (Gy_15_)
Tuleasca et al., 2020 [30]	TN	480	NA	NA	>1 year	87 (Central dose), range 76.1–97.9	range 1535–2675
Warnick et al., 2024 [6]	TN	871	40.1	68, range 23–91	21, range 6–156	80 (Central dose), range 62.5–95	1974, range 1253–2530
Tang et al., 2025 [31]	TN	548	48.5	59, IQR 55–62	84, IQR 58–108	86 (Central dose), IQR 85-88	2719, IQR 2665–2752
Deng et al., 2025 [32]	TN	123	37	63.69 (Mean)	65.5, range 12–147	83 (Central dose)	1968 (Mean)
Tuleasca et al., 2024 [33]	ET	78	61.5	71, range 36–88	12, range 3–36	130 Gy for all cases (Maximal dose)	4612, range 4022–4944

VS, Vestibular schwannoma; AVM, arteriovenous malformation; MBM, melanoma brain metastases; TN, trigeminal neuralgia; ET, essential tremor; BED, biological effective dose; NA, not applicable; IQR, interquartile range.

**Table 2:** Effects of BED/physical dose on treatment outcomes.

Studies	Effects of BED on GKS Efficacy	Effects of BED on GKS Toxicity	Effects of Physical Dose on GKS Efficacy	Effects of Physical Dose on GKS Toxicity
Pituitary adenoma				
Graffeo et al., 2020 [17]	BED was significantly associated with biochemical remission after GKS (HR: 1.01, 95% CI: 1.00–1.02, *p* = 0.02).	Not significantly associated with a new post-GKS hypopituitarism.	Margin dose was not significantly associated with biochemical remission (HR: 1.09, 95% CI: 0.98–1.24, *p* = 0.1).	Not significantly associated with a new post-GKS hypopituitarism.
Balossier et al., 2021 [18]	Higher BED group (>199.7 Gy_2.47_) corresponds to better endocrine remission at 9 years (70.2% vs. 48.2%, *p* > 0.05).	Not significantly associated with a new post-GKS hypopituitarism.	Not significantly associated with endocrine remission.	Not significantly associated with a new post-GKS hypopituitarism.
Dumot et al., 2023 [21]	BED > 170Gy_2.47_ (HR: 2.02, 95% CI: 1.06–3.86, *p* = 0.03) was significantly associated with biochemical remission.	Not significantly associated with a new endocrinopathy.	Margin dose > 22Gy (HR: 2.33, 95% CI: 1.06–5.13, *p* = 0.04) was significantly associated with biochemical remission.	Not significantly associated with a new endocrinopathy.
Balossier et al., 2021 [19]	Higher BED group (>228 Gy_2.47_) corresponds to better endocrine remission at last follow-up (77% vs. 66%, *p* > 0.05).	Not significantly associated with a new post-GKS hypopituitarism.	Not significantly associated with endocrine remission.	Not significantly associated with a new post-GKS hypopituitarism.
Gao et al., 2023 [12]	Higher BED group (>205 Gy_2.47_) associated with to better endocrine remission at 2 years (72.7% vs. 35.1%, *p* = 0.04).	Not significantly associated with a new post-GKS hypopituitarism.	NA	NA
Graffeo et al., 2021 [20]	Not significantly associated with treatment success (growth arrest for non-secreting tumors or biochemical control off medications for secreting tumors)	Mean gland BED (HR: 1.03, 95% CI: 1.02–1.05, *p* < 0.001) was significantly associated with a new post-GKS hypopituitarism. BED based model presented better predictive efficacy of hypopituitarism compared to physical dose-based models.	Not significantly associated with treatment success (growth arrest for non-secreting tumors or biochemical control off medications for secreting tumors)	Mean gland dose (HR: 1.31, 95% CI: 1.16–1.47, *p* < 0.001) was significantly associated with a new post-GKS hypopituitarism.
Meningioma				
Dedeciusova et al., 2022 [22]	Not significantly associated with clinical improvement or tumor control.	No serious adverse events reported.	NA	No serious adverse events reported.
Huo et al., 2022 [23]	Among 354 grade I lesions, higher BED (>50Gy_2.47_) was associated with a lower incidence of local failure (*p* < 0.01).	NA	Not significantly associated with local tumor control.	Margin dose > 12 Gy was significantly associated with radiological and symptomatic edema. (OR: 2.4, 95% CI: 1.2–4.8, *p* = 0.01)
Shaaban et al., 2024 [24]	BED was significantly associated with local tumor control after GKS (HR: 0.96, 95% CI: 0.92–1.00, *p* = 0.03).	NA	Not significantly associated with local tumor control.	NA
Vestibular schwannoma				
Tuleasca et al., 2021 [13]	Increased BED was significantly associated with tumor volume decrease (β: −0.17, *p* < 0.01).	NA	NA	NA
Villafuerte et al., 2021 [25]	Not significantly associated with tumor control.	Not significantly associated with radiologic or symptomatic edema.	Not significantly associated with tumor control.	Prescription dose < 12 Gy was significantly associated with radiologic edema in univariate analysis (OR: 5.48, 95% CI: 1.45–20.7, *p* = 0.01).
Tuleasca et al., 2023, 2024 [26,27]	NA	Tumor BED and mean cochlea BED were significant predictors of hearing decline at various time points after GKS.	NA	Tumor dose was not significantly associated with hearing decline. Mean cochlea dose was significantly associated with hearing decline at 24 months.
Arteriovenous malformation				
Nesvick et al., 2021 [11]	BED > 133 Gy_2.47_ was predictive of AVM obliteration after GKS (HR: 1.52, 95% CI: 1.19–1.95, *p* < 0.01).	NA	Not significantly associated with AVM obliteration.	NA
Tuleasca et al., 2021 [7]	BED was the strongest predictor of obliteration of unruptured AVMs (HR: 1.015, 95% CI: 1.001–1.029, *p* = 0.03).	Not significantly associated with post-GKS complications.	BED-based model presented better predictive efficacy of obliteration compared to margin dose-based models.	Not significantly associated with post-GKS complications.
Grogan et al., 2024 [28]	BED > 180 Gy_2.47_ was predictive of AVM obliteration after GKS (HR: 2.11, 95% CI: 1.30–3.40, *p* < 0.01).	Not significantly associated with radiation-induced changes.	Margin dose > 20 Gy was predictive of AVM obliteration after GKS (HR: 1.90, 95% CI: 1.15–3.13, *p* = 0.01).	Not significantly associated with radiation-induced changes.
Melanoma brain metastases				
Zubatkina et al., 2023 [29]	For various α/β ratios (3,5,10,15), BED was significant associated with local control (α/β = 15, HR: 1.15, 95% CI: 1.04–1.27, *p* < 0.01). BED presented better predictive efficacy of local control compared to margin dose.	Not significantly associated with radiational necrosis.	Margin dose was significant associated with local control (HR: 1.48, 95% CI: 1.05–2.1, *p* = 0.03).	Not significantly associated with radiational necrosis.
Trigeminal neuralgia				
Tuleasca et al., 2020 [30]	Not significantly associated with pain relief.	The incidence of post-GKS hypoesthesia increasing from <5% after a BED of <1800 Gy_2.47_ to 42% after <2600 Gy_2.47_.	Not significantly associated with pain relief.	Not significantly associated with post-GKS hypoesthesia.
Warnick et al., 2024 [6]	BED ≥ 2100 Gy_2.47_ was a significant predictor of initial pain relief for the distal target (HR: 1.46, 95% CI 1.05–2.03, *p* = 0.03).	Not significantly associated with post-GKS sensory dysfunction.	Maximal dose ≥ 85 Gy was a significant predictor of initial pain relief for the proximal target (HR 1.79, 95% CI 1.05–3.05; *p* = 0.03).	Not significantly associated with post-GKS sensory dysfunction.
Tang et al., 2025 [31]	^a^ V%_CN V-BED1000_ was a significant predictor of pain relief (OR: 1.05, 95% CI: 1.04–1.07, *p* < 0.01), quality of life (OR: 1.05, 95% CI: 1.04–1.07, *p* < 0.01) and medication withdrawal (OR: 1.05, 95% CI: 1.04–1.05, *p* < 0.01).	Maximal BED received by brainstem was a significant predictor of complications (OR: 1.06, 95% CI: 1.05–1.06, *p* < 0.01).	Not significantly associated with efficacy outcomes.	Not significantly associated with post-GKS complications.
Deng et al., 2025 [32]	BED was a significant predictor of failure for the distal target (OR: 0.996, 95% CI 0.992–0.999, *p* = 0.02).	BED was a significant predictor of post-GKS complications for the distal target (OR: 1.002, 95% CI 1.00–1.004, *p* = 0.01).	Not significantly associated with efficacy outcomes.	Central dose was a significant predictor of post-GKS complications for the distal target (OR: 1.40, 95% CI 1.10–1.83, *p* = 0.01).
Essential tremor				
Tuleasca et al., 2024 [33]	BED was significantly associated with improvement in the ET rating assessment scale (β = −0.029, *p* = 0.04).	Adverse radiation events were present in 7 out of 78 (8.9%) cases, with a mean BED of 4650 Gy_2.47_. Five cases of transient hemiparesis were with BED > 4500 Gy_2.47_.	NA	NA

^a^ The ratio of volume of the trigeminal nerve portion covered by iso-BED 1000 Gy_2.47_ to volumes of the trigeminal nerve portion within the prescription isodose surface. BED, biological effective dose; GKS, Gamma knife radiosurgery; OR, odds ratio; HR, hazards ration; CI, confident interval, NA, not applicable.

## Data Availability

Data are contained within the article.

## Supplementary Materials

The following supporting information can be downloaded at: https://www.mdpi.com/article/10.3390/jcm15010381/s1, Supplementary Table S1: Adapted Newcastle–Ottawa Scale (NOS) for Observational Studies Included in the Review; Supplementary Table S2: Key BED Thresholds Across GKS Indications; Supplementary methods 1: the adapted version of the Newcastle–Ottawa Scale (NOS); Supplementary methods 2: the BED formulation; Supplementary Table S3: PRISMA 2020 Checklist [42].

## References

[1] Leksell L. (1983). Stereotactic radiosurgery. J. Neurol. Neurosurg. Psychiatry.

[2] Tuleasca C., Régis J., Sahgal A., De Salles A., Hayashi M., Ma L., Martínez-Álvarez R., Paddick I., Ryu S., Slotman B.J. (2019). Stereotactic radiosurgery for trigeminal neuralgia: A systematic review. J. Neurosurg..

[3] Mahajan U.V., Desai A., Shost M.D., Cai Y., Anthony A., Labak C.M., Herring E.Z., Wijesekera O., Mukherjee D., Sloan A.E. (2022). Stereotactic radiosurgery and resection for treatment of multiple brain metastases: A systematic review and analysis. Neurosurg. Focus.

[4] Kondziolka D. (1999). Functional radiosurgery. Neurosurgery.

[5] Marchetti M., Sahgal A., De Salles A.A.F., Levivier M., Ma L., Paddick I., Pollock B.E., Regis J., Sheehan J., Suh J.H. (2020). Stereotactic Radiosurgery for Intracranial Noncavernous Sinus Benign Meningioma: International Stereotactic Radiosurgery Society Systematic Review, Meta-Analysis and Practice Guideline. Neurosurgery.

[6] Warnick R.E., Paddick I., Mathieu D., Adam E., Iorio-Morin C., Leduc W., Hamel A., Johnson S.E., Bydon M., Niranjan A. (2024). The relevance of biologically effective dose for pain relief and sensory dysfunction after Gamma Knife radiosurgery for trigeminal neuralgia: An 871-patient multicenter study. J. Neurosurg..

[7] Tuleasca C., Peciu-Florianu I., Leroy H.A., Vermandel M., Faouzi M., Reyns N. (2021). Biologically effective dose and prediction of obliteration of unruptured arteriovenous malformations treated by upfront Gamma Knife radiosurgery: A series of 149 consecutive cases. J. Neurosurg..

[8] Fowler J.F. (2010). 21 years of biologically effective dose. Br. J. Radiol..

[9] Millar W.T., Hopewell J.W., Paddick I., Lindquist C., Nordstron H., Lidberg P., Garding J. (2015). The role of the concept of biologically effective dose (BED) in treatment planning in radiosurgery. Phys. Med..

[10] Jones B., Hopewell J.W. (2019). Modelling the influence of treatment time on the biological effectiveness of single radiosurgery treatments: Derivation of “protective” dose modification factors. Br. J. Radiol..

[11] Nesvick C.L., Graffeo C.S., Brown P.D., Link M.J., Stafford S.L., Foote R.L., Laack N.N., Pollock B.E. (2021). The Role of Biological Effective Dose in Predicting Obliteration After Stereotactic Radiosurgery of Cerebral Arteriovenous Malformations. Mayo Clin. Proc..

[12] Gao Y., Wang M., Wu Y., Deng H., Xu Y., Ren Y., Wang C., Wang W. (2023). Gamma Knife Radiosurgery for Cushing’s Disease: Evaluation of Biological Effective Dose from a Single-Center Experience. J. Clin. Med..

[13] Tuleasca C., Faouzi M., Maeder P., Maire R., Knisely J., Levivier M. (2021). Biologically effective dose correlates with linear tumor volume changes after upfront single-fraction stereotactic radiosurgery for vestibular schwannomas. Neurosurg. Rev..

[14] Fowler J.F. (1989). The linear-quadratic formula and progress in fractionated radiotherapy. Br. J. Radiol..

[15] Kirkpatrick J.P., Meyer J.J., Marks L.B. (2008). The linear-quadratic model is inappropriate to model high dose per fraction effects in radiosurgery. Semin. Radiat. Oncol..

[16] Hopewell J.W., Millar W.T., Lindquist C. (2012). Radiobiological principles: Their application to γ knife therapy. Prog. Neurol. Surg..

[17] Graffeo C.S., Donegan D., Erickson D., Brown P.D., Perry A., Link M.J., Young W.F., Pollock B.E. (2020). The Impact of Insulin-Like Growth Factor Index and Biologically Effective Dose on Outcomes After Stereotactic Radiosurgery for Acromegaly: Cohort Study. Neurosurgery.

[18] Balossier A., Tuleasca C., Cortet-Rudelli C., Soto-Ares G., Levivier M., Assaker R., Reyns N. (2021). Gamma Knife radiosurgery for acromegaly: Evaluating the role of the biological effective dose associated with endocrine remission in a series of 42 consecutive cases. Clin. Endocrinol..

[19] Balossier A., Tuleasca C., Cortet-Rudelli C., Soto-Ares G., Levivier M., Assaker R., Reyns N. (2021). Gamma Knife surgery for recurrent or persistent Cushing disease: Long-term results and evaluation of biological effective dose in a series of 26 patients. Swiss Med. Wkly..

[20] Graffeo C.S., Perry A., Link M.J., Brown P.D., Young W.F., Pollock B.E. (2021). Biological Effective Dose as a Predictor of Hypopituitarism After Single-Fraction Pituitary Adenoma Radiosurgery: Dosimetric Analysis and Cohort Study of Patients Treated Using Contemporary Techniques. Neurosurgery.

[21] Dumot C., Schlesinger D., Mantziaris G., Dayawansa S., Xu Z., Sheehan J.P. (2023). Role of biological effective dose for prediction of endocrine remission in acromegaly patients treated with stereotactic radiosurgery. Pituitary.

[22] Dedeciusova M., Komarc M., Faouzi M., Levivier M., Tuleasca C. (2022). Tumor control and radiobiological fingerprint after Gamma Knife radiosurgery for posterior fossa meningiomas: A series of 46 consecutive cases. J. Clin. Neurosci..

[23] Huo M., Rose M., van Prooijen M., Cusimano M.D., Laperriere N., Heaton R., Gentili F., Payne D., Shultz D.B., Kongkham P. (2022). Importance of Cobalt-60 Dose Rate and Biologically Effective Dose on Local Control for Intracranial Meningiomas Treated with Stereotactic Radiosurgery. Neurosurgery.

[24] Shaaban A., Pham D., Tos S.M., Mantziaris G., Schlesinger D., Sheehan J.P. (2024). Biological effective dose as a predictor of local tumor control in stereotactic radiosurgery treated parasellar meningioma patients. J. Neurooncol..

[25] Villafuerte C.J., Shultz D.B., Laperriere N., Gentili F., Heaton R., van Prooijen M., Cusimano M.D., Hodaie M., Schwartz M., Berlin A. (2021). Radiation Dose Rate, Biologically Effective Dose, and Tumor Characteristics on Local Control and Toxicity After Radiosurgery for Acoustic Neuromas. World Neurosurg..

[26] Tuleasca C., Toma-Dasu I., Duroux S., Starnoni D., George M., Maire R., Daniel R.T., Patin D., Schiappacasse L., Dasu A. (2023). The Relevance of Biologically Effective Dose for Hearing Preservation After Stereotactic Radiosurgery for Vestibular Schwannomas: A Retrospective Longitudinal Study. Neurosurgery.

[27] Tuleasca C., Toma-Dasu I., Duroux S., George M., Maire R., Daniel R.T., Patin D., Schiappacasse L., Dasu A., Faouzi M. (2024). Impact of the Mean Cochlear Biologically Effective Dose on Hearing Preservation After Stereotactic Radiosurgery for Vestibular Schwannoma: A Retrospective Longitudinal Analysis. Neurosurgery.

[28] Grogan D., Dumot C., Tewari A., Mantziaris G., Dayawansa S., Schlesinger D., Sheehan J. (2024). Biologically Effective Dose and Prediction of Obliteration of Arteriovenous Malformations in Pediatric Patients Treated by Gamma Knife Radiosurgery. Neurosurgery.

[29] Zubatkina I., Toma-Dasu I., Dasu A., Levivier M., Tuleasca C., Ivanov P. (2024). Clinically Driven Alpha/Beta Ratios for Melanoma Brain Metastases and Investigation of Biologically Effective Dose as a Predictor for Local Control After Radiosurgery: A Proof of Concept in a Retrospective Longitudinal Series of 274 Consecutive Lesions. Neurosurgery.

[30] Tuleasca C., Paddick I., Hopewell J.W., Jones B., Millar W.T., Hamdi H., Porcheron D., Levivier M., Régis J. (2020). Establishment of a Therapeutic Ratio for Gamma Knife Radiosurgery of Trigeminal Neuralgia: The Critical Importance of Biologically Effective Dose Versus Physical Dose. World Neurosurg..

[31] Tang K., Zhang N., Yuan X., Chu L., Qian Z., Li Y. (2025). Radiosurgical Biologically Effective Dose on Trigeminal Root Division and Section for Outcomes of Idiopathic Trigeminal Neuralgia Type 1: A Multicentre Retrospective Cohort Study. World Neurosurg..

[32] Deng H., Gao Y., Wu Y., Wang M., Xiao L., Chen R., Zhang Z., Pan W., Wang W. (2025). Differential impact of biologically effective dose in distal versus proximal gamma knife targets for trigeminal neuralgia. Front. Neurol..

[33] Tuleasca C., Carey G., Barriol R., Touzet G., Dubus F., Luc D., Carriere N., Reyns N. (2024). Impact of biologically effective dose on tremor decrease after stereotactic radiosurgical thalamotomy for essential tremor: A retrospective longitudinal analysis. Neurosurg. Rev..

[34] Plataniotis G.A., Dale R.G. (2009). Biologically effective dose-response relationship for breast cancer treated by conservative surgery and postoperative radiotherapy. Int. J. Radiat. Oncol. Biol. Phys..

[35] Moreno A.C., Fellman B., Hobbs B.P., Liao Z., Gomez D.R., Chen A., Hahn S.M., Chang J.Y., Lin S.H. (2020). Biologically Effective Dose in Stereotactic Body Radiotherapy and Survival for Patients with Early-Stage NSCLC. J. Thorac. Oncol..

[36] Farooqi A., Ludmir E.B., Mitchell K.G., Antonoff M.B., Tang C., Lee P., Chang J., Elamin Y., Gomez D.R., Gandhi S.J. (2021). Increased biologically effective dose (BED) to the primary tumor is associated with improved survival in patients with oligometastatic NSCLC. Radiother. Oncol..

[37] Kocher M., Treuer H., Voges J., Hoevels M., Sturm V., Müller R.P. (2000). Computer simulation of cytotoxic and vascular effects of radiosurgery in solid and necrotic brain metastases. Radiother. Oncol..

[38] Leith J.T., Cook S., Chougule P., Calabresi P., Wahlberg L., Lindquist C., Epstein M. (1994). Intrinsic and extrinsic characteristics of human tumors relevant to radiosurgery: Comparative cellular radiosensitivity and hypoxic percentages. Acta Neurochir. Suppl..

[39] Barzaghi L.R., Albano L., Scudieri C., Gigliotti C.R., Del Vecchio A., Mortini P. (2021). Factors affecting long-lasting pain relief after Gamma Knife radiosurgery for trigeminal neuralgia: A single institutional analysis and literature review. Neurosurg. Rev..

[40] Lee J.Y., Sandhu S., Miller D., Solberg T., Dorsey J.F., Alonso-Basanta M. (2015). Higher dose rate Gamma Knife radiosurgery may provide earlier and longer-lasting pain relief for patients with trigeminal neuralgia. J. Neurosurg..

[41] Pop L.A., Millar W.T., van der Plas M., van der Kogel A.J. (2000). Radiation tolerance of rat spinal cord to pulsed dose rate (PDR-) brachytherapy: The impact of differences in temporal dose distribution. Radiother. Oncol..

[42] Page M.J., McKenzie J.E., Bossuyt P.M., Boutron I., Hoffmann T.C., Mulrow C.D., Shamseer L., Tetzlaff J.M., Akl E.A., Brennan S.E. (2021). The PRISMA 2020 statement: An updated guideline for reporting systematic reviews. BMJ.

