# Supplementary material for: The Role of Biological Effective Dose in Gamma Knife Radiosurgery: A Systematic Review Across Multiple Indications
Source: J Clin Med. 2026 Jan 5;15(1):381. doi: 10.3390/jcm15010381 (PMC12786754; doi:10.3390/jcm15010381)
Supplement: Supplementary file 1 [file jcm-15-00381-s001.zip › jcm-4014669-supplementary.pdf]

Supplementary methods 1 the adapted version of the Newcastle–Ottawa Scale (NOS)

This review applied an adapted version of the Newcastle–Ottawa Scale (NOS) to evaluate methodological quality and risk of bias in the included observational studies. The scale consisted of three domains—Selection, Comparability, and Outcome—with a maximum score of 8 points. Higher scores indicate higher study quality.

Domain 1. Selection (0–4 points)

Representativeness of the cohort

1 point: Clearly representative patient cohort.

0 point: Selected, non-representative sample.

Ascertainment of exposure (GKS parameters including dose and BED)

1 point: Exposure clearly described with standardized methods.

0 point: Exposure description incomplete or unclear.

Documentation of baseline disease characteristics

1 point: Comprehensive baseline data reported.

0 point: Incomplete baseline information.

Clarity in describing BED model and calculation

1 point: Biexponential repair model explicitly described.

0 point: Insufficient description of BED computation.

Domain 2. Comparability (0–2 points)

Adjustment or control for key confounders

2 points: Major confounders controlled.

1 point: Partial adjustment.

0 point: No adjustment.

Domain 3. Outcome (0–2 points)

Outcome assessment and definition

1 point: Objective, clearly defined outcome measures.

0 point: Outcome definition unclear.

Adequacy of follow-up

1 point: Sufficient follow-up duration for the disease (⩾24 months).

0 point: Insufficient or poorly reported follow-up.

Supplementary Table S1 Adapted Newcastle–Ottawa Scale (NOS) for Observational Studies Included in the Review

Study (Author, Year)	Selection (0–4)	Comparability (0–2)	Outcome (0–2)	Total Score (0–8)	Quality Category*
Graffeo et al., 2020[17]	4	2	2	8	High
Balossier et al., 2021[18]	4	2	2	8	High
Dumot et al., 2023[21]	4	2	2	8	High
Balossier et al., 2021[19]	4	2	2	8	High
Gao et al., 2023[12]	3	1	1	4	Moderate
Graffeo et al., 2021[20]	3	2	2	7	High
Dedeciusova et al., 2022[22]	4	2	2	8	High
Huo et al., 2022[23]	4	2	2	8	High
Shaaban et al., 2024[24]	4	2	2	8	High
Tuleasca et al., 2021[13]	4	2	2	8	High
Villafuerte et al., 2021[25]	3	2	2	7	High
Tuleasca et al., 2023, 2024[26,27]	4	2	2	8	High
Nesvick et al., 2021[11]	4	2	2	8	High
Tuleasca et al., 2021[7]	4	1	2	7	High
Grogan et al., 2024[28]	4	2	1	7	High
Zubatkina et al., 2023[29]	3	2	2	7	High
Tuleasca et al., 2020[30]	3	1	1	5	Moderate
Warnick et al., 2024[6]	4	2	2	8	High
Tang et al., 2025[31]	4	2	2	8	High
Deng et al., 2025[32]	4	2	2	8	High
Tuleasca et al., 2024[33]	4	2	1	7	High

*High quality: 6–8; Moderate quality: 4–5; Low quality: ≤3

Supplementary Table S2 Key BED Thresholds Across GKS Indications

Indications	Reported BED Threshold	Associated Outcome	Direction of Effect
Pituitary adenoma – Acromegaly	BED >170-200 $Gy_{2.47}$	Biochemical remission	Higher BED associated with improved hormonal control
Meningioma	BED >50-68 $Gy_{2.47}$	Local tumor control	Higher BED predicts improved local control
Arteriovenous malformation	BED >133-180 $Gy_{2.47}$	Obliteration	Higher BED associated with higher obliteration rates
Trigeminal neuralgia	BED >1850-2100 $Gy_{2.47}$ for distal target	Initial pain relief	Higher BED associated with better initial pain relief
Melanoma brain metastasis	BED >49 Gy_{15}	Tumor volume decrease	Higher BED associated with quicker decrease in volume first month after GKRS
Essential tremor	BED >4350 $Gy_{2.47}$ and <4600 $Gy_{2.47}$	The Essential Tremor Rating Assessment Scale	Increasing BED was associated with improvement in the ET rating scale

Supplementary methods 2 the BED formulation

$$BED = \frac{nd}{1+c}\left[1 + \frac{(n-1)d}{k} f(\mu_1 T) + \frac{d}{k} f(\mu_1 t)\right] + \frac{cnd}{1+c}\left[1 + \frac{(n-1)d}{k} f(\mu_2 T) + \frac{d}{k} f(\mu_2 t)\right]$$

where *n* represents the number of shots for a single treatment and *nd* is the total physical dose for a single treatment. *c* is the partition coefficient associated with the fast/slow two-phase repair rate of sublethal radiation damage. $k=\alpha/\beta$ is a tissue-specific constant. The α/β ratio refers to the single dose at which the lethal and sublethal injury of irradiation have the same cytotoxic effect. $Gy_{2.47}$ is the unit of BED with an α/β ratio of 2.47 Gy. The parameter μ refers to sublethal radiation damage repair rates under protracted irradiation. μ_1 represents the fast phase repair rate, and μ_2 is the slow rate. *T* is the total time spent on a single treatment, and *t* is the average time spent on each shot. $f(\mu t)$ is a function associated with sublethal damage repair and time[20]. The function is as follows:

$$f(\mu T) = \frac{2}{\mu T}\left[1 - \frac{(1 - e^{-\mu T})}{1 - \mu T}\right]$$

Supplementary Table S3 PRISMA 2020 Checklist

Section and Topic	Item #	Checklist item	Location where item is reported
**TITLE**			
Title	1	Identify the report as a systematic review.	1
**ABSTRACT**			
Abstract	2	See the PRISMA 2020 for Abstracts checklist.	1
**INTRODUCTION**			
Rationale	3	Describe the rationale for the review in the context of existing knowledge.	2
Objectives	4	Provide an explicit statement of the objective(s) or question(s) the review addresses.	2
**METHODS**			
Eligibility criteria	5	Specify the inclusion and exclusion criteria for the review and how studies were grouped for the syntheses.	2
Information sources	6	Specify all databases, registers, websites, organisations, reference lists and other sources searched or consulted to identify studies. Specify the date when each source was last searched or consulted.	2
Search strategy	7	Present the full search strategies for all databases, registers and websites, including any filters and limits used.	2
Selection process	8	Specify the methods used to decide whether a study met the inclusion criteria of the review, including how many reviewers screened each record and each report retrieved, whether they worked independently, and if applicable, details of automation tools used in the process.	3
Data collection process	9	Specify the methods used to collect data from reports, including how many reviewers collected data from each report, whether they worked independently, any processes for obtaining or confirming data from study investigators, and if applicable, details of automation tools used in the process.	3
Data items	10a	List and define all outcomes for which data were sought. Specify whether all results that were compatible with each outcome domain in each study were sought (e.g. for all measures, time points, analyses), and if not, the methods used to decide which results to collect.	3
	10b	List and define all other variables for which data were sought (e.g. participant and intervention characteristics, funding sources). Describe any assumptions made about any missing or unclear information.	3
Study risk of bias assessment	11	Specify the methods used to assess risk of bias in the included studies, including details of the tool(s) used, how many reviewers assessed each study and whether they worked independently, and if applicable, details of automation tools used in the process.	3
Effect measures	12	Specify for each outcome the effect measure(s) (e.g. risk ratio, mean difference) used in the synthesis or presentation of results.	3
Synthesis methods	13a	Describe the processes used to decide which studies were eligible for each synthesis (e.g. tabulating the study intervention characteristics and comparing against the planned groups for each synthesis (item #5)).	3
	13b	Describe any methods required to prepare the data for presentation or synthesis, such as handling of missing summary statistics, or data conversions.	3
	13c	Describe any methods used to tabulate or visually display results of individual studies and syntheses.	3
	13d	Describe any methods used to synthesize results and provide a rationale for the choice(s). If meta-analysis was performed, describe the model(s), method(s) to identify the presence and extent of statistical heterogeneity, and software package(s) used.	3
	13e	Describe any methods used to explore possible causes of heterogeneity among study results (e.g. subgroup analysis, meta-regression).	NA
	13f	Describe any sensitivity analyses conducted to assess robustness of the synthesized results.	NA
Reporting bias assessment	14	Describe any methods used to assess risk of bias due to missing results in a synthesis (arising from reporting biases).	3
Certainty assessment	15	Describe any methods used to assess certainty (or confidence) in the body of evidence for an outcome.	3

Supplementary Table S3 PRISMA 2020 Checklist

Section and Topic	Item #	Checklist item	Location where item is reported
**RESULTS**			
Study selection	16a	Describe the results of the search and selection process, from the number of records identified in the search to the number of studies included in the review, ideally using a flow diagram.	3
	16b	Cite studies that might appear to meet the inclusion criteria, but which were excluded, and explain why they were excluded.	3
Study characteristics	17	Cite each included study and present its characteristics.	Table1
Risk of bias in studies	18	Present assessments of risk of bias for each included study.	Table S1
Results of individual studies	19	For all outcomes, present, for each study: (a) summary statistics for each group (where appropriate) and (b) an effect estimate and its precision (e.g. confidence/credible interval), ideally using structured tables or plots.	Table2
Results of syntheses	20a	For each synthesis, briefly summarise the characteristics and risk of bias among contributing studies.	7-9
	20b	Present results of all statistical syntheses conducted. If meta-analysis was done, present for each the summary estimate and its precision (e.g. confidence/credible interval) and measures of statistical heterogeneity. If comparing groups, describe the direction of the effect.	7-9
	20c	Present results of all investigations of possible causes of heterogeneity among study results.	11
	20d	Present results of all sensitivity analyses conducted to assess the robustness of the synthesized results.	NA
Reporting biases	21	Present assessments of risk of bias due to missing results (arising from reporting biases) for each synthesis assessed.	7
Certainty of evidence	22	Present assessments of certainty (or confidence) in the body of evidence for each outcome assessed.	7-9
**DISCUSSION**			
Discussion	23a	Provide a general interpretation of the results in the context of other evidence.	9-10
	23b	Discuss any limitations of the evidence included in the review.	10-11
	23c	Discuss any limitations of the review processes used.	10-11
	23d	Discuss implications of the results for practice, policy, and future research.	11
**OTHER INFORMATION**			
Registration and protocol	24a	Provide registration information for the review, including register name and registration number, or state that the review was not registered.	3
	24b	Indicate where the review protocol can be accessed, or state that a protocol was not prepared.	3
	24c	Describe and explain any amendments to information provided at registration or in the protocol.	3
Support	25	Describe sources of financial or non-financial support for the review, and the role of the funders or sponsors in the review.	11-12
Competing interests	26	Declare any competing interests of review authors.	11-12
Availability of data, code and other materials	27	Report which of the following are publicly available and where they can be found: template data collection forms; data extracted from included studies; data used for all analyses; analytic code; any other materials used in the review.	11-12

From: Page MJ, McKenzie JE, Bossuyt PM, Boutron I, Hoffmann TC, Mulrow CD, et al. The PRISMA 2020 statement: an updated guideline for reporting systematic reviews. BMJ 2021;372:n71. doi: 10.1136/bmj.n71
